# Deciphering Proteomic Expression in Inflammatory Disorders: A Mass Spectrometry Exploration Comparing Infectious, Noninfectious, and Traumatic Brain Injuries in Human Cerebrospinal Fluid

**DOI:** 10.1089/neur.2024.0050

**Published:** 2024-09-20

**Authors:** Philip Dyhrfort, Caroline Lindblad, Anna Widgren, Johan Virhammar, Fredrik Piehl, Jonas Bergquist, Faiez Al Nimer, Elham Rostami

**Affiliations:** ^1^ Department of Medical Sciences, Uppsala University, Uppsala, Sweden.; ^2^ Department of Neurosurgery, Uppsala University Hospital, Uppsala, Sweden.; ^3^ Department of Clinical Neuroscience, Karolinska Institutet, Stockholm, Sweden.; ^4^ Department of Clinical Neurosciences, Addenbrooke’s Hospital, Cambridge University, Turku, Finland.; ^5^ Department of Chemistry—BMC, Analytical Chemistry and Neurochemistry, Uppsala University, Uppsala, Sweden.; ^6^ Department of Neurology, Uppsala University Hospital, Uppsala, Sweden.; ^7^ Center for Neurology, Academic Specialist Center, Stockholm, Sweden.; ^8^ Department of Neuroscience, Karolinska Institutet, Stockholm, Sweden.

**Keywords:** central nervous system, encephalitis, fluidic protein biomarker, human studies, inflammation, mass-spectrometry, neurointensive care, traumatic brain injury

## Abstract

The central nervous system (CNS) evokes a complex inflammatory response to injury. Inflammatory cascades are present in traumatic, infectious, and noninfectious disorders affecting the brain. It contains a mixture of pro- and anti-inflammatory reactions involving well-known proteins, but also numerous proteins less explored in these processes. The aim of this study was to explore the distinct inflammatory response in traumatic brain injury (TBI) compared with other CNS injuries by utilization of mass-spectrometry. In total, 56 patients had their cerebrospinal fluid (CSF) analyzed with the use of mass-spectrometry. Among these, CSF was collected via an external ventricular drain (EVD) from *n* = 21 patients with acute TBI. The resulting protein findings were then compared with CSF obtained by lumbar puncture from *n* = 14 patients with noninfectious CNS disorders comprising relapsing–remitting multiple sclerosis, anti-*N*-methyl-d-aspartate-receptor encephalitis, acute disseminated encephalomyelitis, and *n* = 14 patients with progressive multifocal leukoencephalopathy, herpes simplex encephalitis, and other types of viral meningitis. We also utilized *n* = 7 healthy controls (HCs). In the comparison between TBI and noninfectious inflammatory CNS disorders, concentrations of 55 proteins significantly differed between the groups. Among them, 23 and 32 proteins were up- and downregulated, respectively, in the TBI group. No proteins were uniquely identified in either group. In the comparison of TBI and HC, 51 proteins were significantly different, with 24 and 27 proteins being up- and downregulated, respectively, in TBI. Two proteins (fibrinogen gamma chain and transketolase) were uniquely identified in all samples of the TBI group. Also in the last comparison, TBI versus infectious inflammatory CNS disorders, 51 proteins differed between the two groups, with 19 and 32 proteins being up- and downregulated, respectively, in TBI, and no unique proteins being identified. Due to large discrepancies between the groups compared, the following proteins were selected for further deeper analysis among those being differentially regulated: APOE, CFB, CHGA, CHI3L1, C3, FCGBP, FGA, GSN, IGFBP7, LRG1, SERPINA3, SOD3, and TTR. We found distinct proteomic profiles in the CSF of TBI patients compared with HC and different disease controls, indicating a specific interplay between inflammatory factors, metabolic response, and cell integrity. In relation to primarily infectious or inflammatory disorders, unique inflammatory pathways seem to be engaged, and could potentially serve as future treatment targets.

## Introduction

The central nervous system (CNS) mounts a complex inflammatory response to injury. Following both CNS traumatic and nontraumatic insults, resident immune cells, such as microglia and astrocytes, become activated.^1^ This activation triggers the release of proinflammatory mediators, including cytokines, chemokines, and reactive oxygen species. The immune response serves to clear damaged cells, debris, and pathogens, and to initiate and sustain a process of tissue repair. However, dysregulation of this inflammatory process can contribute to secondary tissue damage and neurodegeneration.^2^ Trauma triggers inflammation via dying cells and leaking intracellular material by so-called damage-associated molecular patterns, DAMPs, which are potent triggers of the innate immune system.^3^ In contrast, both CNS infections and autoimmune disorders are also characterized by prominent involvement of the adaptive immune system, with involvement of both cellular and humoral immune components.^4,5^ Detailed mapping of the characteristics of inflammatory responses being mounted in different contexts has implications for understanding the diverse pathological processes and designing targeted therapeutic interventions.^6–9^

Cerebrospinal fluid (CSF) biomarkers have long played an important diagnostic role in autoimmune conditions such as multiple sclerosis (MS), but more lately also serving as markers of disease activity. Hence, studies have shown that interleukin (IL-) 10 and C-X-C motif chemokine ligand (CXCL) 13 levels are predictive of further disease activity in relapsing–remitting MS (RRMS),^10,11^ while CSF CC chemokine ligand (CCL)11 becomes upregulated in secondary progressive multiple sclerosis,^12^ while also being correlated to cognitive decline and psychiatric disorder.^13,14^ Levels of IL-6 predict both age of onset of MS and exhibit increased levels in MS patients’ serum.^15^ CSF biomarker studies in herpes simplex encephalitis (HSE) show an association between extensive CNS inflammation and long-term neurocognitive outcomes.^16–18^ Moreover, a longitudinal study demonstrated that while some proinflammatory and anti-inflammatory cytokines such as interferon-gamma, tumor necrosis factor-alpha, IL-6, IL-1β, and IL-10 became upregulated early, other cytokines (e.g., CCL17, CCL21-27, and CXCL12-13) peaked later, thus indicating a temporal dependency of the inflammatory response.^18^ Taken together, several biomarkers, for example, IL-6, IL-10, and CXCL-13 can be found and have sparked interest in all the abovementioned inflammatory disorders, and also notably in TBI.^19–22^ In fact, IL-6 is also pivotal in neurotrauma. IL-6 mediates the acute-phase response^23^ and regulates lymphocyte and monocyte differentiation processes.^24^ In experimental neurotrauma, IL-6 depletion yields a subdued glial cell response and diminishes sequelae,^25^ and conversely, IL-6 overexpression precipitates neurodegenerative consequences.^26,27^ Clinically, IL-6 levels correlate proportionately with the gravity of the traumatic insult^28^ and seem to be an indicator of clinical outcome.^29^ In the clinical domain, a recurrent motif emerges—the ascent of IL-6 aligns with the exacerbation of TBI. In contrast, the temporal dynamics of IL-10 in clinical studies on TBI remain inconclusive^21,22,30^ and despite numerous attempts no consistent link between IL-10 levels and clinical outcomes has been established.^31–33^ Broadly, IL-10 enhancement emerges as a promising therapeutic target, enhancing neurological outcomes following TBI.^34^ TBI research related to the complement system and its inhibition in the early phases after trauma might have neuroprotective benefits,^35,36^ while others show increased blood–brain barrier (BBB) damage with activation.^37^ Taken together, discernment of the inflammatory response for each separate disorder has been made, but there is still a gap in the knowledge about similarities and dissimilarities between various inflammatory CNS disorders.

Above, we highlight shared inflammatory cues across various types of CNS insults, indicating that various CNS disorders share common neuroinflammatory response pathways, while each disorder also holds a distinct neuroinflammatory phenotype. This could tentatively be of importance for improved pathophysiological understanding and disease-specific treatment development. To study this, we sought to utilize mass spectrometry to delineate and compare fluid proteomic expressions in the CSF and blood across nontraumatic inflammatory disorders of infectious and noninfectious origin and compare these with the inflammatory response in TBI patients. Specifically, we compared noninfectious inflammatory disorders RRMS, anti-*N*-methyl-d-aspartate-receptor encephalitis (NMDAE), and acute disseminated encephalomyelitis (ADEM), and infectious inflammatory disorders (HSE and progressive multifocal leukoencephalopathy [PML]) with patients suffering from acute severe TBI. The core aim of this study was to identify TBI-unique inflammatory proteins.

## Methods

This was a prospective, observational investigation undertaken among patients at the Karolinska University Hospital and Karolinska Institutet in Stockholm, Sweden, between 2003 and 2018. In total, *n* = 56 patients were included in this study. Of these, *n* = 21 patients had suffered a TBI. In contrast, *n* = 16 patients, including *n* = 6 with RRMS, *n* = 6 with NMDAE, and *n* = 4 with ADEM, had suffered a noninfectious inflammatory disease of the CNS. In total, *n* = 14 patients were categorized as CNS infections, specifically *n* = 6 patients with PML, *n* = 6 patients with HSE, and *n* = 2 patients with viral meningitis. In total, *n* = 7 healthy controls (HCs) were included between 2013 and 2018.

The study was approved by the Swedish Ethics Review Authority 2005/1526/31/2, 2014/1201–31-1 and 2009/2107–31/2. Written informed consent was obtained by all patients or, during neurointensive care (NIC), patient relatives.

## Group Division

We divided the data set into *n* = 4 analytical groups. Group 1 constituted patients suffering from TBI. Group 2 entailed noninfectious acute neuroinflammatory disorders such as RRMS, NDMAE, and ADEM. Group 3 was utilized as a control group and consisted of healthy persons. Group 4 comprised patients suffering from acute CNS infections (PML, HSE, and other types of viral meningitis).

### Patient population, management, and sample acquisition from the TBI-group

TBI patients were included if they were adult subjects (18–75 years) who had sustained a severe TBI (classified as Glasgow Coma Scale [GCS] 3–8 upon hospital admission or else GCS >8 score, but with a clinically deemed strong risk of deterioration) in need of neurointensive care unit (NICU) treatment at the Karolinska University Hospital with implantation of invasive intracranial monitoring. The following four factors constituted the exclusion criteria: (i) devastating prognosis before NICU arrival, (ii) penetrating head injury, (iii) comatose due to other factors than TBI, (iv) a preexisting chronic condition that precluded subsequent follow-up or other reasons precluding adequate follow-up. The local NICU management protocol of severe TBI has been detailed elsewhere.^38^ In summary, the Karolinska University Hospital follows an intracranial pressure (ICP)-driven strategy, aligning with the guidelines set forth by the Brain Trauma Foundation.^39^ ICP is monitored either through a closed external ventricular drain (EVD) (Medtronic, USA), or an intraparenchymal pressure monitor (Codman & Shurtleff Inc. Raynham, MA, USA, or Rehau AG+CO, Rehay, Germany). Data obtained from monitors were retrieved via the TBI database at the Karolinska University Hospital. Additional clinical variables, radiological variables, as well as outcome score data were collected prospectively.^40^ Glasgow outcome score (GOS) was collected at 6–12 months following hospital discharge via a questionnaire or a physical meeting at the outpatient clinic. CSF sample collection from the TBI patients was collected from an EVD. The median time for acquiring the CSF samples in this group was around 6.0 days and the mean time was 5.2 days, with the longest sampling collecting time being 11 days and the shortest 1 day. Samples were stored locally at 4°C in median 1 day (0–1), until delivery to a local biobank, where samples were vertically incubated for 30 min before centrifugation for 15 min at 2000 *g*, aliquoted, and stored at −80°C until further analysis.

### Patient population, management, and sample acquisition from groups 2, 3, and 4

The patients constituting groups 2, 3, and 4 were recruited during the time frame described above and samples were handled in a similar manner as outlined above, with the difference that the primary caretaking clinic was the Neurology Department at Karolinska University Hospital. Importantly, the CSF in these groups was obtained via lumbar punctures. The cohort with MS was, during the sampling time, not treated with any immune-modulation treatment, which could affect proteomic analysis. Notably, one of the patients suffering from NMDAE (group 2) had received rituximab, and one patient was treated with corticosteroids before sampling. Also, all patients with ADEM were on corticosteroids during the time of sampling.

### Proteomic analysis

Aliquots corresponding to 150 µL of initial CSF samples were taken out for analyzing. Before analyzing, the whole volume of each CSF sample (150 µL) was filtered through a 0.22 μm cellulose acetate spin filter (Agilent Technologies, Palo Alto, CA, USA) by centrifugation at 14 000 × *g* for 2 min. Afterward, an aliquot of 145 µL of each CSF sample was loaded onto the Multiple Affinity Removal System (MARS Hu-14) (Agilent Technologies, Palo Alto, CA) cartridge and the flow-through (FT) fraction was collected by centrifugation for 2 min at 100 × *g*. Two successive wash steps with 400 µL of MARS-7 Buffer A were carried out to obtain maximum yield. The FT and wash (W) fractions were combined. The spin cartridge was washed with 2.5 ml of MARS-7 Buffer B to remove bound proteins and was then re-equilibrated with Buffer A. The remaining fractions (FT+W) were dried using SpeedVac (Thermo Fisher Scientific, Waltham, MA). The proteins were reduced, alkylated, and in-solution digested by trypsin according to the standard operating procedure. The collected peptide filtrate was cleaned by C18-spin columns (Thermo Fisher) and then vacuum centrifuged to dryness using a SpeedVac system. Dried peptides were resolved in 30 μL of 0.1% formic acid and further diluted 2 times, nano-LC-MS/MS. The peptides were separated in reverse phase on a C18-column with a 90-min gradient and electrosprayed on-line to a Q-Exactive Plus mass spectrometer (Thermo Finnigan). Tandem mass spectrometry was performed applying HCD.

## Protein Characterization

Database searches were performed using the software Proteome Discoverer ver. 1.4. The search was set toward proteins from the *Homo sapiens* proteome extracted from UniProt in 2020. The search parameters were set to Taxonomy: *Homo sapiens*, Enzyme: Trypsin. Fixed modification was carbamidomethyl (C), and variable modifications were oxidation (M) and deamidated (NQ). The search criteria for protein identification were set to at least two matching peptides. With the use of the websites UniProt and GeneCards, the regulated group of proteins was scanned and conveniently distributed according to their main function in the following groups: Cell adhesion, Enzymatic, Hormone-related proteins, Metabolism, Immune response, and Miscellaneous.

### Data and statistical analysis

For demographic and clinical data, continuous variables are presented as mean (standard deviation) if normally distributed and else median (interquartile range). For mass spectrometry data, further raw-data processing was necessary. The RAW-data files were quantitatively analyzed by the quantification software MaxQuant 1.5.3.30 (Max-Planck Institute of Biochemistry). The results of all fractions were combined to a total label-free intensity analysis for each sample, which was multiple testing corrected using the false discovery rate. Next, peptides without missing observations were examined across groups 1–4. As group 1 was the study group of interest (TBI patients), calculations were done as a series of Student’s *t tests* between group 1 and group n, where n could take the value 2, 3, or 4. No multiple-testing correction was done after this, but proteins were only considered significant/regulated if they exhibited a *p* value ≤0.05 and an absolute and relative difference of ∼Δ20% compared with group n.

Graphical presentation was conducted using R (The Comprehensive R Archive Network, version 4.3.1)^41^ via the interface RStudio (2023.9.0.463). In addition to base R, we also utilized the packages tidyverse,^42^ gridExtra,^43^ and magrittr.^44^

## Results

### Group 1 (TBI)

The cohort of severe TBI patients consisted of 21 patients (17 males and 4 females), with a mean age of 52 ± 8 years (Table 1). All patients except *n* = 2 had an admission GCS score of less than eight, and nine of the admitted patients did not have a free airway. All except one suffered from hypoxia.

**Table 1.** Demography, Admission Variables, and Clinical Outcome in Group 1 (TBI)

Case no.	1	2	3	4	5	6	7	8	9	10	11	12	13	14	15	16	17	18	19	20	21
Gender	M	M	M	M	M	M	M	M	M	M	M	W	W	M	M	M	W	W	M	M	M
Age	64	60	60	50	50	41	41	49	49	64	64	49	49	39	58	58	57	57	44	38	60
GCS Adm.	3	3	3	11	11	3	3	6	6	5	5	7	7	7	3	3	4	4	3	7	7
Free Airway	−	+	+	+	+	−	−	−	−	−	−	+	+	+	+	+	+	+	NA	−	−
Pupils	1	0	0	0	0	0	0	0	0	0	0	0	0	NA	0	0	1	1	1	0	0
Rotterdam CT Score	4	5	5	4	4	6	6	6	6	4	4	3	3	3	3	3	3	3	2	3	3
Marshall CT Score	6	4	4	3	3	6	6	6	6	6	6	6	6	6	2	2	2	2	2	2	2
Progressive Hematoma	−	+	+	−	−	−	−	+	+	+	+	−	−	−	+	+	−	−	−	−	−
Head−AIS	5	5	5	4	4	5	5	5	5	5	5	4	4	4	4	4	4	4	4	5	5
NISS	57	57	57	34	34	75	75	75	75	57	57	29	29	34	34	34	48	48	34	57	57
GOS	4	1	1	5	5	4	4	3	3	1	1	4	4	3	4	4	3	3	4	3	3

The table includes characteristics of the 21 patients. Age span reached from 38 to 64 years. Glasgow Coma Scale (GCS) was noted at admission (3–15). Pupils (0–2, 0 normal, 1 unilateral unresponsive, 2 bilateral unresponsive). Rotterdam CT Score (1–6). Marshall CT Score (1–6). Head-Abbreviated Injury Scale (AIS) (1–6). New Injury Severity Score (NISS) (Max 75). Glasgow Outcome Score (GOS) was recorded at follow-up at around 6–12 months after the time of injury (1–5).

### Group 2 (RRMS, NMDAE, and ADEM), group 3 (HC), group 4 (PML, HSE, and other viral meningitis)

We entitle noninfectious CNS inflammations (entailing RRMS, NMDAE, and ADEM) “Group 2.” Group 2 consisted of 14 patients (six males and eight females), with a mean age of 45 (range, 18–64). Group 3 here defined as a control group consisted of seven healthy individuals (three males and four females), with a mean age of 41 ± 5 SD. Group 4, that is, infectious, nontraumatic CNS neuroinflammations, constituted 14 patients (3 males and 11 females) of mean age 51 (range, 26–70).

### Identified biomarkers and regulations between groups

**FIG. 1.**
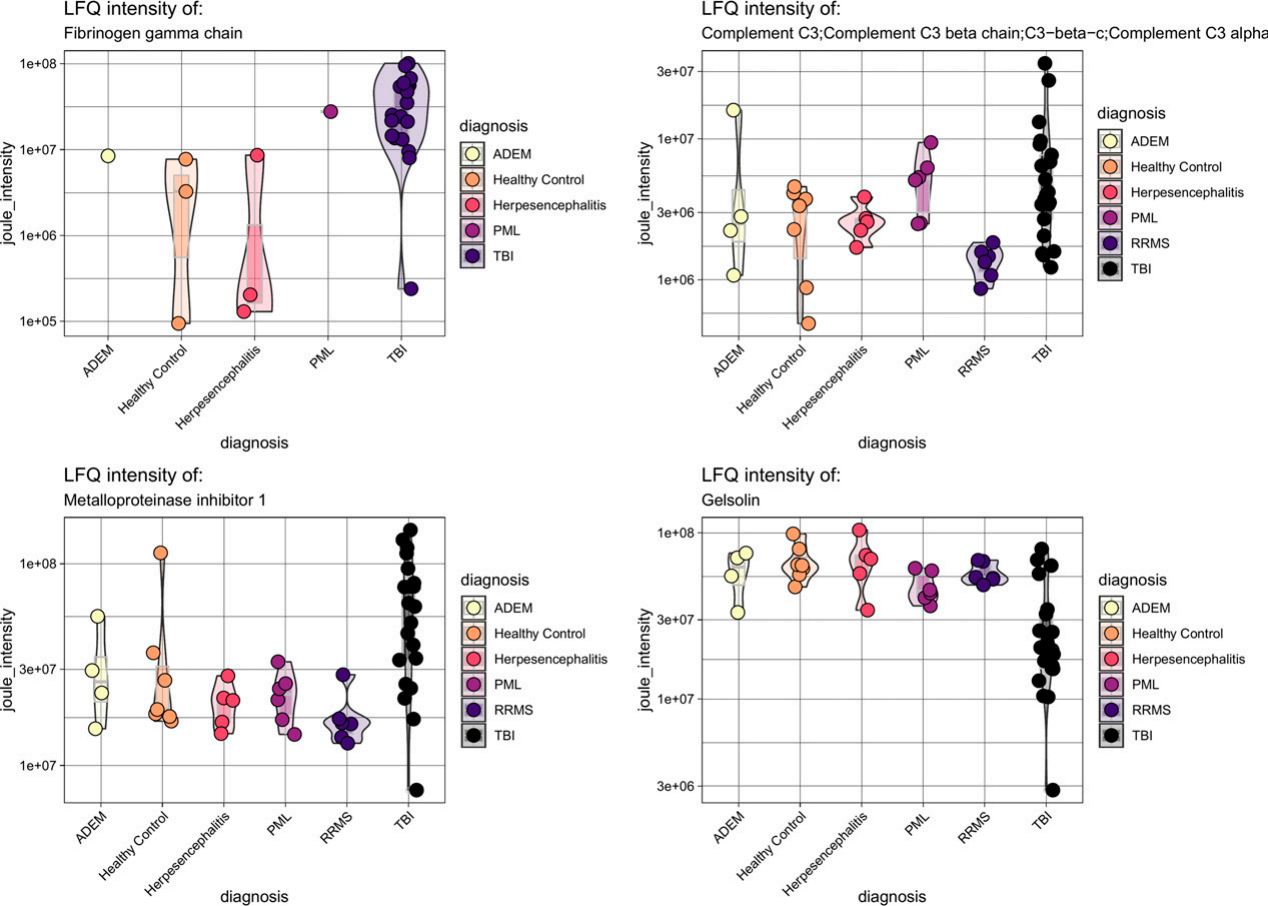
Representative proteins illustrating protein-level homo- and heterogeneity across neuroinflammatory diagnoses. The figure depicts four selected proteins (FGA, C3, TIMP1, and Gelsolin). The X-axis constitutes different diagnoses ADEM, herpes encephalitis, healthy controls, PML, RRMS (except in Table 1 where FGA was not among the regulated proteins), and TBI, while the Y-axis shows the joule intensity. Each dot represents one patient, and the coloring is unique for each disease. FGA, fibrinogen alpha chain; C3, complement C3; TIMP, metalloproteinase inhibitor; ADEM, acute disseminated encephalomyelitis; PML, progressive multifocal leukoencephalopathy; RRMS, relapsing–remitting multiple sclerosis; TBI, traumatic brain injury.

**FIG. 2.**
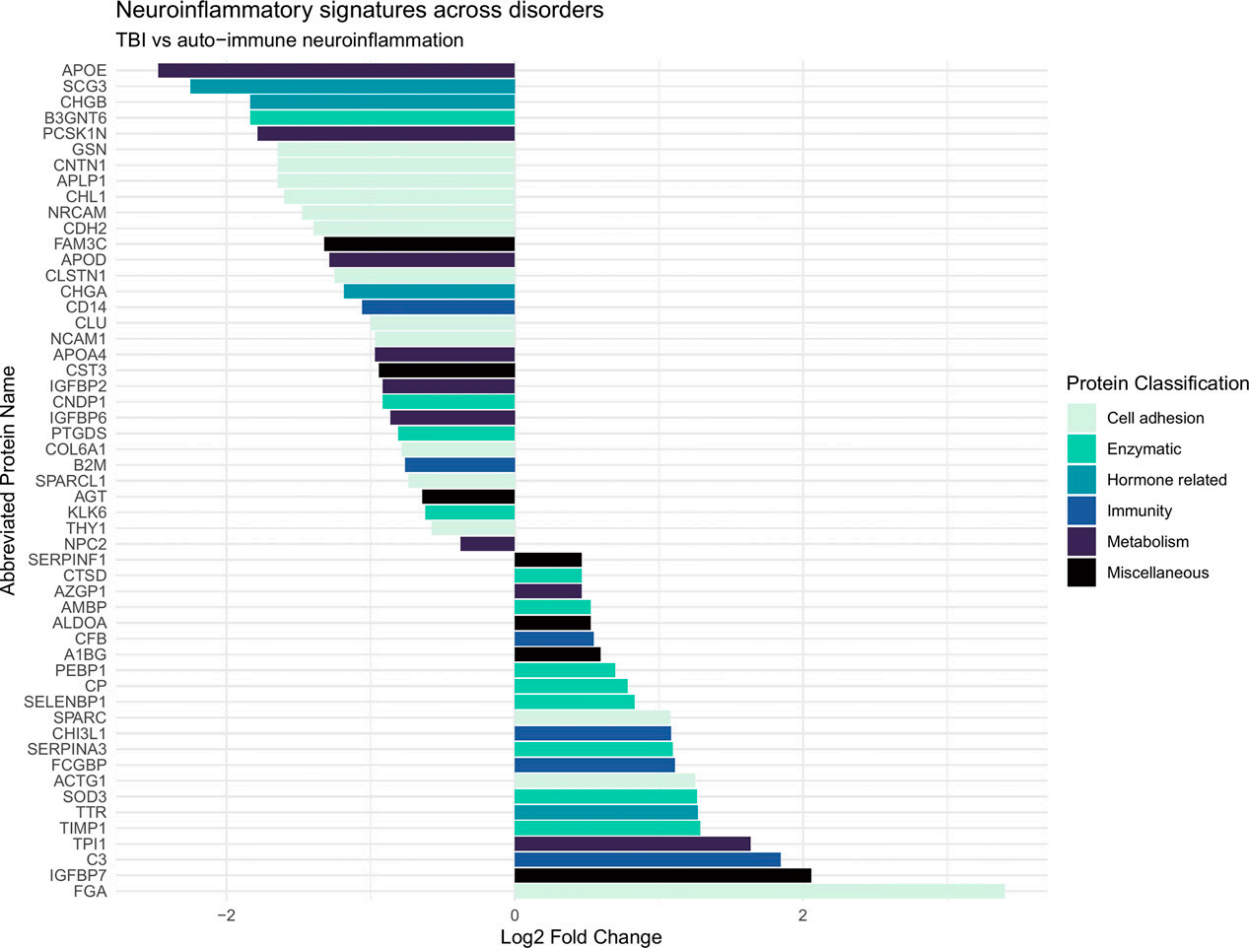
Visual display of the regulated proteins in the comparison of TBI versus the autoimmune inflammatory disorder group, their LFQ intensity, and their respective protein classification. The X-axis illustrates the log2 fold change, both positive and negative numbers. The Y-axis depicts the list of proteins with their corresponding abbreviated name. The color coding is based on the protein group classification made in this study. TBI, traumatic brain injury; LFQ, label free quantitation.

**FIG. 3.**
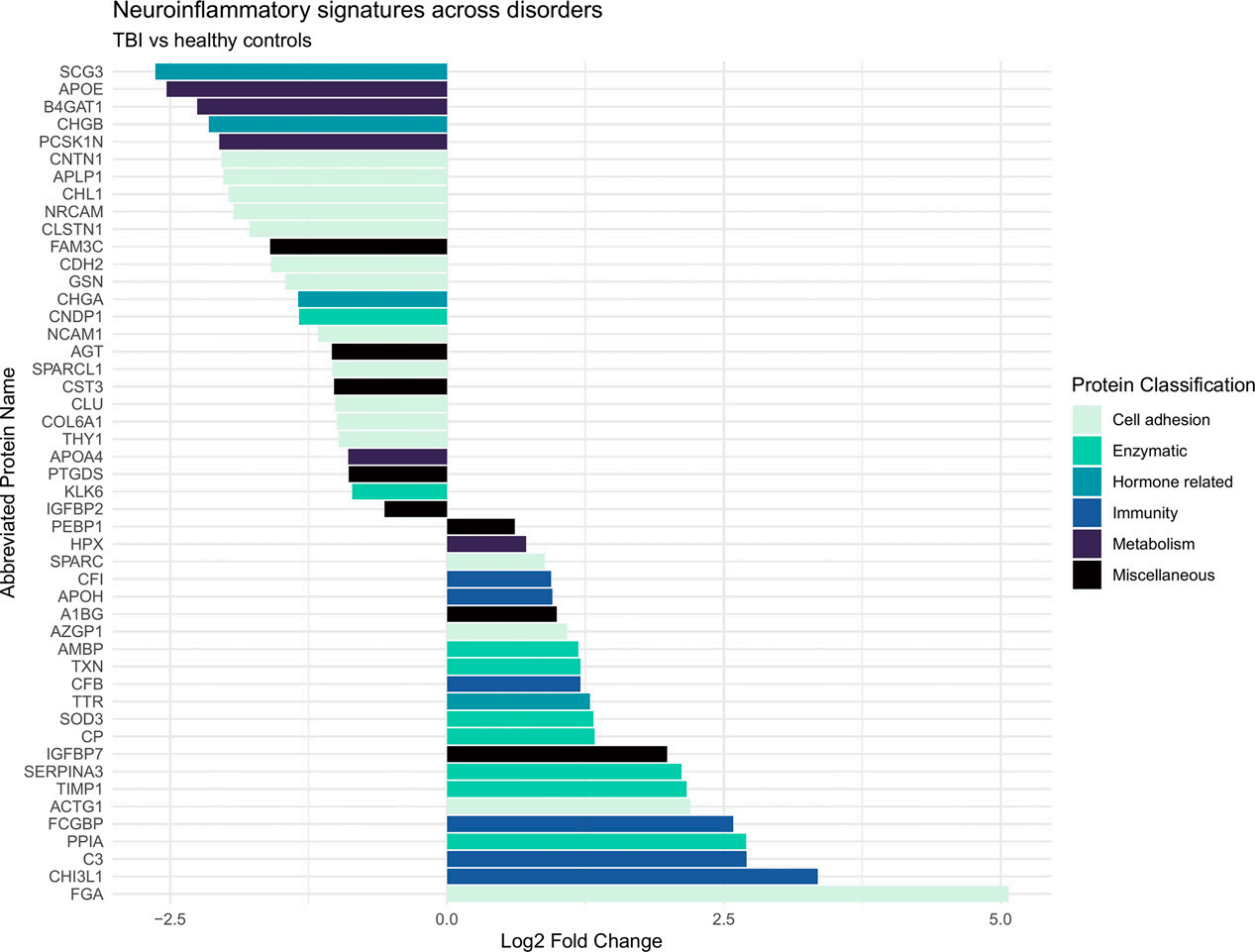
Visual display of the regulated proteins in the comparison of TBI versus the healthy control group, their LFQ intensity, and their respective protein classification. The X-axis illustrates the log2 fold change, both positive and negative numbers. The Y-axis depicts the list of proteins with their corresponding abbreviated name. The color coding is based on the protein group classification made in this study. TBI, traumatic brain injury; LFQ, label free quantitation.

**FIG. 4.**
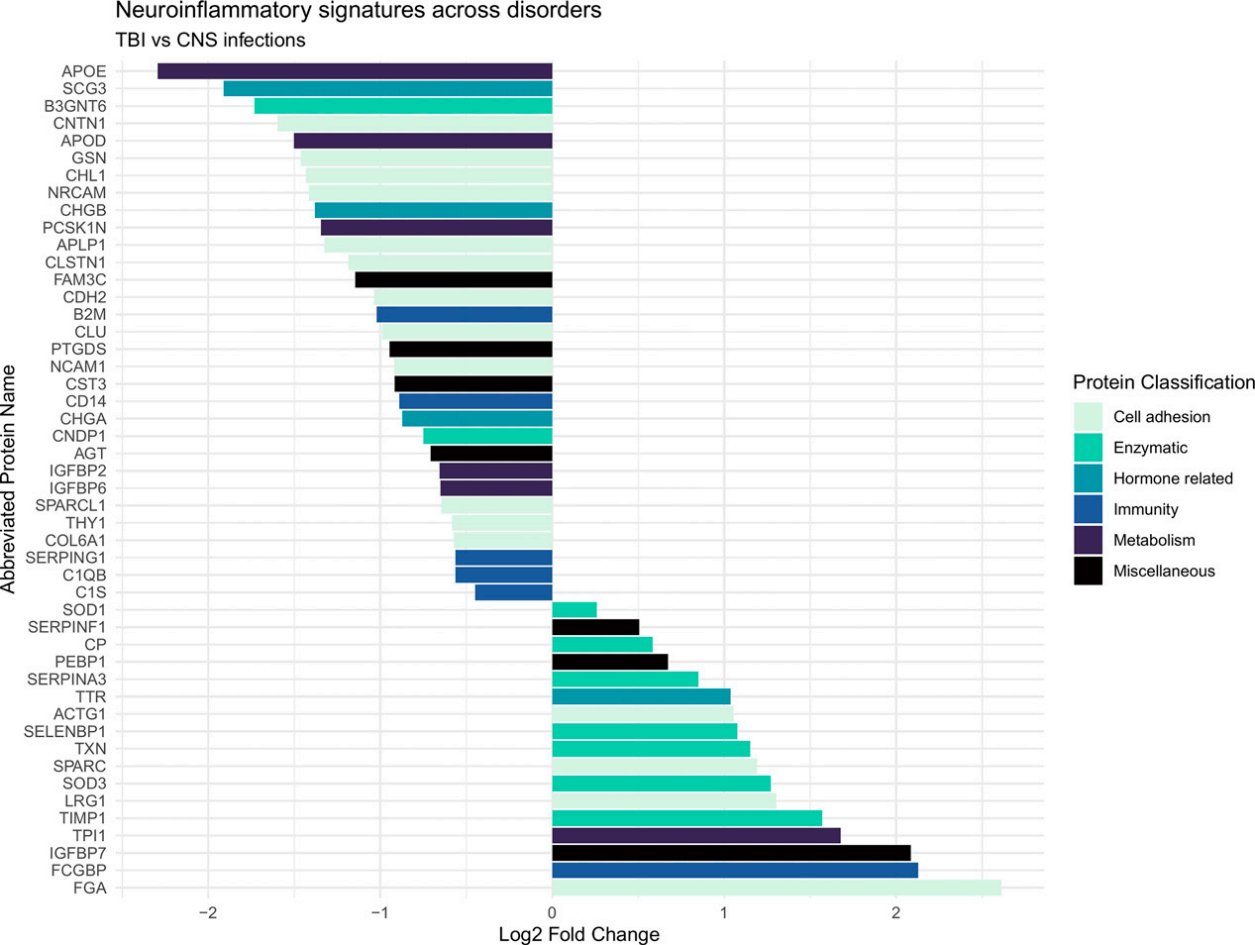
Visual display of the regulated proteins in the comparison of TBI versus the CNS infection group, their LFQ intensity, and their respective protein classification. The X-axis illustrates the log2 fold change, both positive and negative numbers. The Y-axis depicts the list of proteins with their corresponding abbreviated name. The color coding is based on the protein group classification made in this study. CNS, central nervous system; LFQ, label free quantitation.

The number of identified proteins in the samples against the *Homo sapiens* proteome is given in Tables 2–4. In total, we identified *n* = 1486 proteins identified in all 56 samples. The label free quantitation (LFQ) intensity is shown for a selected group of biomarkers (FGA, gelsolin, TIMP1, C3), in Figure 1, to illustrate the level of homo- and heterogeneity across neuroinflammation. The displayed proteins were chosen because they have created interest in previous research as well as being on the list of regulated proteins. Figures 2, 3, and 4 display all the regulated proteins in the three different group comparisons, their LFQ intensity, and their respective protein classification.

**Table 2.** Regulated Proteins Between Group 1 and Group 2

No.	Short name	Full name	UnitProtKB ID	Regulated	Classification
1	FGA	Fibrinogen alpha chain	P02671	++	Cell adhesion
2	IGFBP7	Insulin-like growth factor-binding protein 7	Q16270	++	Cell adhesion Hormone related
3	C3	Complement C3	P01024	++	Immunity
4					
5	TPI1	Triosephosphate isomerase	P60174	++	Metabolism
6	TIMP1	Metalloproteinase inhibitor 1	P01033	++	Enzymatic
5	TTR	Transthyretin	P02766	++	Hormone related
8	SOD3	Superoxide dismutase	P08294	++	Enzymatic
9	ACTG1	Actin gamma 1	P63261	++	Cell adhesion
10	FCGBP	IgGFc-binding protein	Q9Y6R7	++	Immunity
11	SERPINA3	Serine proteinase inhibitor	P01011	++	Enzymatic
12	CHI3L1	Chitinase-3-like protein 1	P36222	++	Immunity
13	SPARC	SPARC	P09486	++	Cell adhesion
14	SELENBP1	Selenium-binding protein 1	Q13228	+	Enzymatic
15	CP	Ceruloplasmin	P00450	+	Enzymatic
16	PEBP1	Phosphatidylethanolamine-binding protein 1	P30086	+	Enzymatic
17	A1BG	Alpha-1B-glycoprotein	P04217	+	Miscellaneous
18	CFB	Complement factor B	P00751	+	Immunity
19	ALDOA	Aldolase	P04075	+	Metabolism, Enzymatic
20	AMBP	Alpha-1-microglobulin	P02760	+	Enzymatic
21	AZGP1	Zinc-alpha-2-glycoprotein	P25311	+	Metabolism
22	CTSD	Cathepsin D	P07339	+	Enzymatic
23	SERPINF1	Pigment epithelium-derived factor	P36955	+	Miscellaneous
24	NPC2	Epididymal secretory protein E1	P61916	−	Metabolism
25	THY1	Thy-1 membrane glycoprotein	P04216	−	Cell adhesion
26	KLK6	Kallikrein-6	Q92876	−	Enzymatic
27	AGT	Angiotensinogen	P01019	−	Miscellaneous
28	SPARCL1	SPARC-like protein 1	Q14515	−	Cell adhesion
29	B2M	Beta-2-microglobulin	P61769	−	Immunity
30	COL6A1	Collagen alpha-1(VI) chain	P12109	−	Cell adhesion
31	PTGDS	Prostaglandin-H2 D-isomerase	P41222	−	Enzymatic
32	IGFBP6	Insulin-like growth factor-binding protein 6	P24592	−	Metabolism
33	IGFBP2	Insulin-like growth factor-binding protein 2	P18065	−	Metabolism
34	CNDP1	Beta-Ala-His dipeptidase	Q96KN2	−	Enzymatic
35	CST3	Cystatin-C	P01034	−	Miscellaneous
36	APOA4	Apolipoprotein A-IV	P06727	−	Metabolism
37	NCAM1	Neural cell adhesion molecule 1	P13191	−	Cell adhesion
38	CLU	Clusterin	P10909	−	Cell adhesion
39	CD14	Monocyte differentiation antigen CD14	P08571	−−	Immunity
40	CHGA	Chromogranin-A	P10645	−−	Hormone related
41	CLSTN1	Calsyntenin-1	Q94985	−−	Cell adhesion
42	APOD	Apolipoprotein D	P05090	−−	Metabolism
43	FAM3C	Protein FAM3C	Q92520	−−	Miscellaneous
44	CDH2	Cadherin-2	P19022	−−	Cell adhesion
45	NRCAM	Neuronal cell adhesion molecule	Q92823	−−	Cell adhesion
46					
47	CHL1	Neural cell adhesion molecule L1-like protein	O00533	−−	Cell adhesion
48	CNTN1	Contactin-1	Q12860	−−	Cell adhesion
49	APLP1	Amyloid-like protein 1	P51693	−−	Cell adhesion
50	GSN	Gelsolin	P06396	−−	Cell adhesion
51	PCSK1N	Protein convertase subtilisin	Q9UHG2	−−	Metabolism
52	B3GNT6	Beta-1,4-glucuronyltransferase 1	Q6ZMB0	−−	Enzymatic
53	CHGB	Secretogranin-1	P05060	−−	Hormone related
54	SCG3	Secretogranin-3	Q8WXD2	−−	Hormone related
55	APOE	Apolipoprotein E	P02649	−−	Metabolism

+ indicates upregulated between one- and twofold. ++ indicates upregulated above twofold. − indicates downregulated between one- and twofold. − − indicates downregulated between one- and twofold. For convenience, the proteins are divided in six different groups regarding their classification: Cell adhesion, Enzymatic, Hormone-related proteins, Immunity, Metabolism, and Miscellaneous.

### Comparison group 1 (TBI) versus group 2 (RRMS, NMDAE, and ADEM)

In total, we quantified 86 proteins, of which 55 proteins were differentially expressed between the groups. Among these, 23 proteins were upregulated among TBI patients and 32 proteins were downregulated. No proteins were uniquely identified in group 1 and group 2. Two peptides could not be identified.

Among the 23 upregulated proteins, 13 proteins were upregulated more than twofold. Cell adhesion: FGA 10.56 times and IGFBP7 4.16 times. Enzymatic: TIMP1 2.44 times, SOD3 2.40 times, and SERPINA3 2.14 times. Hormone related: TTR 2.41 times. Immunity: CHI3L1 2.12 times, C3 3.59 times, and FCGBP 2.16 times (Table 2).

Among the 32 downregulated proteins, 17 proteins were downregulated more than twofold. Cell adhesion: Gelsolin 0.32 times. Hormone related: CHGA 0.44 times. Metabolism: APOE 0.18 times (Table 2).

### Comparison of group 1 (TBI) versus group 3 (HC)

In total, we quantified *n* = 88 proteins, of which *n* = 51 were significantly altered between groups 1 and 3. Among these, 24 proteins were upregulated in group 1 and 27 proteins were downregulated in the TBI-group. Two proteins (fibrinogen gamma chain and transketolase) were uniquely identified in all the samples of group 1 but were never found in group 3. No proteins were uniquely identified in group 3. Three peptides could not be categorized.

Among the upregulated proteins, 18 were being upregulated more than twofold. Cell adhesion: FGA 33.62 times and IGFBP7 3.97 times. Enzymatic: TIMP1 4.48 times, SOD3 2.50 times, and SERPINA3 4.33 times. Hormone related: TTR 2.45 times. Immunity: C3 6.52 times and FCGBP 6.00 times (Table 3).

**Table 3.** Regulated Proteins Between Groups 1 and 3

No.	Short name	Full name	UnitProtKB ID	Regulated	Classification
1	FGA	Fibrinogen alpha chain	P02671	++	Cell adhesion
2	CHI3L1	Chitinase-3-like protein 1	P36222	++	Immunity
3	C3	Complement C3	P01024	++	Immunity
4	PPIA	Peptidyl-prolyl cis-trans isomerase A	P62937	++	Enzymatic
5	FCGBP	IgGFc-binding protein	Q9Y6R7	++	Immunity
6	ACTG1	Actin gamma 1	P63261	++	Cell adhesion
7	TIMP1	Metalloproteinase inhibitor 1	P01033	++	Enzymatic
8	SERPINA3	Serine proteinase inhibitor	P01011	++	Enzymatic
9	IGFBP7	Insulin-like growth factor-binding protein 7	Q16270	++	Cell adhesion, Hormone related
10					
11	CP	Ceruloplasmin	P00450	++	Enzymatic
12	SOD3	Extracellular superoxide dismutase	P08294	++	Enzymatic
13	TTR	Transthyretin	P02766	++	Hormone related
14					
15	CFB	Complement factor B	P00751	++	Immunity
16	TXN	Thioredoxin	P10599	++	Enzymatic
17	AMBP	a-1-microglobulin	P02760	++	Enzymatic
18	AZGP1	Zinc-alpha-2-glycoprotein	P25311	++	Cell adhesion
19	A1BG	Alpha-1B-glycoprotein	P04217	+	Miscellaneous
20	APOH	Beta-2-glycoprotein 1	P02749	+	Immunity
21	CFI	Complement factor I	P05156	+	Immunity
22	SPARC	SPARC	P09486	+	Cell adhesion
23	HPX	Hemopexin	P02790	+	Metabolism
24	PEBP1	Phosphatidylethanolamine-binding protein 1	P30086	+	Miscellaneous
25	IGFBP2	Insulin-like growth factor-binding protein 2	P18065	−	Cell adhesion, Hormone related
26	KLK6	Kallikrein-6	Q92876	−	Enzymatic
27	PTGDS	Prostaglandin-H2 D-isomerase	P41222	−	Miscellaneous
28	APOA4	Apolipoprotein A-IV	P06727	−	Metabolism
29	THY1	Thy-1 membrane glycoprotein	P04216	−	Cell adhesion
30	COL6A1	Collagen alpha-1(VI) chain	P12109	−	Cell adhesion
31	CLU	Clusterin	P10909	−	Cell adhesion
32	CST3	Cystatin-C	P01034	−−	Miscellaneous
33	SPARCL1	SPARC-like protein 1	Q14515	−−	Cell adhesion
34	AGT	Angiotensinogen	P01019	−−	Miscellaneous
35	NCAM1	Neural cell adhesion molecule 1	P13191	−−	Cell adhesion
36	CNDP1	Beta-Ala-His dipeptidase	Q96KN2	−−	Enzymatic
37	CHGA	Chromogranin-A	P10645	−−	Hormone related
38	GSN	Gelsolin	P06396	−−	Cell adhesion
39					
40	CDH2	Cadherin-2	P19022	−−	Cell adhesion
41	FAM3C	Protein FAM3C	Q92520	−−	Miscellaneous
42	CLSTN1	Calsyntenin-1	Q94985	−−	Cell adhesion
43	NRCAM	Neuronal cell adhesion molecule	Q92823	−−	Cell adhesion
44	CHL1	Neural cell adhesion molecule L1-like protein	O00533	−−	Cell adhesion
45	APLP1	Amyloid-like protein 1	P51693	−−	Cell adhesion
46	CNTN1	Contactin-1	Q12860	−−	Cell adhesion
47	PCSK1N	Protein convertase subtilisin	Q9UHG2	−−	Metabolism
48	CHGB	Secretogranin-1	P05060	−−	Hormone related
49	B4GAT1	Beta-1,4-glucuronyltransferase 1	O43505	−−	Metabolism
50	APOE	Apolipoprotein E	P02649	−−	Metabolism
51	SCG3	Secretogranin-3	Q8WXD2	−−	Hormone related

Regulated proteins between groups 1 and 3. + indicates upregulated between one- and twofold. ++ indicates upregulated above twofold. – indicates downregulated between one- and twofold. − − indicates downregulated between one- and twofold. For convenience, the proteins are divided in six different groups regarding their classification: Cell adhesion, Enzymatic, Hormone-related proteins, Immunity, Metabolism, and Miscellaneous.

Among the 27 downregulated proteins, 20 were downregulated at least twofold. Cell adhesion: Gelsolin 0.32 times. Hormone related: CHGA 0.39 times. Metabolism: APOE 0.17 times (Table 3).

### Comparison of group 1 (TBI) versus group 4 (PML, HSE, and other viral meningitis)

Eighty-eight proteins were quantified, 51 proteins were significantly regulated between groups 1 and 4. Among them, 19 proteins were upregulated in group 1 and 32 proteins were downregulated in the TBI-group. No proteins were uniquely identified in groups 1 and 4. Three peptides could not be categorized.

Nineteen proteins were upregulated at least onefold, with 13 of them being upregulated more than twofold. Three peptides could not be categorized. Cell adhesion: FGA 6.11 times, LRG1 2.47, and IGFBP7 4.24. Enzymatic: TIMP1 2.97 times, SOD3 2.41 times, and SERPINA3 1.80 times. Hormone related: TTR 2.05 times. Immunity: FCGBP 4.37 (Table 4).

Thirty-two proteins were downregulated, with 16 of them being downregulated more than twofold. Cell adhesion: Gelsolin 0.36 times. Hormone related: CHGA 0.55 times. Metabolism: APOE 0.20. Immunity: FCGBP 4.37 (Table 4).

**Table 4.** Regulated Proteins Between Group 1 and Group 4

No.	Short name	Full name	UnitProtKB ID	Regulated	Classification
1	FGA	Fibrinogen alpha chain	P02671	++	Cell adhesion
2	FCGBP	IgGFc-binding protein	Q9Y6R7	++	Immunity
3	IGFBP7	Insulin-like growth factor-binding protein 7	Q16270	++	Cell adhesion, Hormone related
4	TPI1	Triosephosphate isomerase	P60174	++	Metabolism
5	TIMP1	Metalloproteinase inhibitor 1	P01033	++	Enzymatic
6					
7	LRG1	Leucine rich alpha-2-glycoprotein 1	P02750	++	Cell adhesion
8	SOD3	Superoxide dismutase	P08294	++	Enzymatic
9	SPARC	SPARC	P09486	++	Cell adhesion
10	TXN	Thioredoxin	P10599	++	Enzymatic
11	SELENBP1	Selenium-binding protein 1	Q13228	++	Enzymatic
12	ACTG1	Actin gamma 1	P63261	++	Cell adhesion
13	TTR	Transthyretin	P02766	++	Hormone related
14	SERPINA3	Alpha-1-antichymotrypsin	P01011	+	Enzymatic
15					
16	PEBP1	Phosphatidylethanolamine-binding protein 1	P30086	+	Miscellaneous
17	CP	Ceruloplasmin	P00450	+	Enzymatic
18	SERPINF1	Pigment epithelium-derived factor	P36955	+	Miscellaneous
19	SOD1	Superoxide dismutase	P00441	+	Enzymatic
20	C1S	Complement C1s subcomponent	P09871	−	Immunity
21	C1QB	Complement C1q subcomponent subunit B	P02746	−	Immunity
22	SERPING1	Plasma protease C1 inhibitor	P05155	−	Immunity
23	COL6A1	Collagen alpha-1(VI) chain	P12109	−	Cell adhesion
24	THY1	Thy-1 membrane glycoprotein	P04216	−	Cell adhesion
25	SPARCL1	SPARC-like protein 1	Q14515	−	Cell adhesion
26	IGFBP6	Insulin-like growth factor-binding protein 6	P24592	−	Metabolism
27	IGFBP2	Insulin-like growth factor-binding protein 2	P18065	−	Metabolism
28	AGT	Angiotensinogen	P01019	−	Miscellaneous
29	CNDP1	Beta-Ala-His dipeptidase	Q96KN2	−	Enzymatic
30	CHGA	Chromogranin-A	P10645	−	Hormone related
31	CD14	Monocyte differentiation antigen CD14	P08571	−	Immunity
32	CST3	Cystatin-C	P01034	−	Miscellaneous
33	NCAM1	Neural cell adhesion molecule 1	P13191	−	Cell adhesion
34	PTGDS	Prostaglandin-H2 D-isomerase	P41222	−	Miscellaneous
35	CLU	Clusterin	P10909	−	Cell adhesion
36	B2M	Beta-2-microglobulin	P61769	−−	Immunity
37	CDH2	Cadherin-2	P19022	−−	Cell adhesion
38	FAM3C	Protein FAM3C	Q92520	−−	Miscellaneous
39	CLSTN1	Calsyntenin-1	Q94985	−−	Cell adhesion
40	APLP1	Amyloid-like protein 1	P51693	−−	Cell adhesion
41				−−	
42	PCSK1N	Protein convertase subtilisin	Q9UHG2	−−	Metabolism
43	CHGB	Secretogranin-1	P05060	−−	Hormone related
44	NRCAM	Neuronal cell adhesion molecule	Q92823	−−	Cell adhesion
45	CHL1	Neural cell adhesion molecule L1-like protein	O00533	−−	Cell adhesion
46	GSN	Gelsolin	P06396	−−	Cell adhesion
47	APOD	Apolipoprotein D	P05090	−−	Metabolism
48	CNTN1	Contactin-1	Q12860	−−	Cell adhesion
49	B3GNT6	Beta-1,4-glucuronyltransferase 1	Q6ZMB0	−−	Enzymatic
50	SCG3	Secretogranin-3	Q8WXD2	−−	Hormone related
51	APOE	Apolipoprotein E	P02649	−−	Metabolism

Regulated proteins between Group 1 and Group 4. + indicates upregulated between one- and twofold. ++ indicates upregulated above twofold. – indicates downregulated between one- and twofold. − − indicates downregulated between one- and twofold. For convenience, the proteins are divided in six different groups regarding their classification: Cell adhesion, Enzymatic, Hormone-related proteins, Immunity, Metabolism, and Miscellaneous.

## Discussion

Unlike most existing TBI proteomic studies in the literature, this work leveraged multiple comparator disease control groups, aside of HCs, to address features specific for neurotrauma. Interestingly, several protein biomarkers showed a distinct up-/downregulation in both infectious and noninfectious CNS disorders compared with TBI. This included, for example FGA, FCGBP, and APOE. In contrast, other proteins were uniquely found in merely one of the group comparisons, such as CIS and C1QB. Collectively, these findings demonstrate a unique TBI proteomic fingerprint compared with traditional conditions characterized by CNS neuroinflammation. The pattern detected for different protein group domains and the suggested relevance for understanding of underlying disease processes are discussed in the following sections.

### Cell adhesion

The precursor protein fibrinogen alpha chain (FGA) was the most upregulated biomarker in all three group comparisons. FGA is a subunit of the coagulation factor fibrinogen and involved in coagulation. Following BBB disruption, fibrinogen gains access to the brain, but there is also a smaller amount of intrinsic CNS production.^45^ Fibrinogen, which precedes fibrin formation, interacts with the neurovascular unit’s various cellular elements. This interaction directly impacts inflammatory, degenerative, and regenerative mechanisms in cases of neurological injury, as it binds to receptors on neuronal cells.^46,47^ Importantly, TBI patients are expected to have larger amounts of blood intracranially due to structural BBB damage. In two randomized controlled trials (CRASH-2 and CRASH-3), findings indicate that the antifibrinolytic agent tranexamic acid has properties that might lower mortality rates in some TBI patients. This reduction is achieved by inhibiting hyperfibrinolytic disseminated intravascular coagulation, particularly in cases characterized by elevated levels of fibrin degradation products (FDP) and D-dimer.^48,49^ The findings with much higher levels in the TBI group point to a much more disrupted BBB in these patients.

The glycoprotein leucine-rich alpha-2-glycoprotein 1 (LRG1) mediates protein–protein interaction and contributes to cell adhesion and was observed to be upregulated about 2.5 times in the TBI group compared with the infectious disease group. Following cerebral ischemia–reperfusion injury, LRG1 is recognized as a potential signaling molecule. LRG1 is linked to increased brain infarct volume, enhanced autophagy, and apoptosis of brain cells, thereby contributing to the aggravation of cerebral ischemia–reperfusion injury.^50,51^ Experimental rodent work has shown that LRG1 silencing may alleviate intracranial status following sepsis-associated encephalitis.^52^ This indicates that LRG1 is a potentially important molecule in both ischemic-related injuries and infection-driven inflammation in the brain.

The involvement of insulin-like growth factor binding protein, IGFBP7, in the pathogenesis of brain injury is unclear. When endothelial cells (ECs) are exposed to IGFBP7, it triggers the formation of stress fibers and disrupts VE-cadherin-mediated junctions. This disruption culminates in heightened vascular permeability, as demonstrated by increased breakdown of the BBB, suggesting a potential role of IGFBP7 in this process.^53^ Furthermore, when brain ECs are stimulated with IGFBP7, there is an upregulation of E-selectin—a pivotal molecule in the recruitment of immune cells.^54^ This observation suggests that IGFBP7 potentially plays a role in regulating neuroinflammation in response to brain injury.

A study conducted by Wang et al. unveils significant molecular changes in ECs and highlights IGFBP7 as a potential biomarker for vasculature in the context of brain injury. The contradictory impact of IGFBP7 on angiogenesis in various systems might be contingent upon environmental cues within the microenvironment, including the unique regional composition of the extracellular matrix.^55^ This study found that IGFBP7 upregulated about 4 times in each of our group comparisons, highlighting the role of angiogenesis particularly in TBI-related injury.

Gelsolin (GSN), calcium-dependent actin-binding, mediating cell shape and motility, was significantly downregulated in TBI patients compared with all other groups. A study showed that GSN had a biphasic decreasing trend during the first 7 days and a multivariate analysis showed it to be a predictor of 1-month mortality^56^ and 1-year morbidity/mortality.^57^ Studies have demonstrated a similar pattern in various organs during acute illness or trauma, but this was not observed in our study.^58^

### Enzymatic

Metalloproteinase inhibitor 1(TIMP1), a metallopeptidase, was markedly elevated in the three comparisons, about four times when compared with HCs. The protein is believed to exert a protective role for the BBB and vascular integrity.^59^ The findings in our study show a marked increase for the TBI group pointing out a more extensive BBB damage where TIMP1 is upregulated to protect the integrity of damaged BBB. Exploring treatments directed at TIMP1 and its associated downstream elements holds promise as an innovative approach to safeguard the integrity of the BBB in disorders affecting the CNS, particularly in the context of TBI.^59^

We observed a 1.5 to 2.5 times upregulation of the antioxidant, superoxide dismutase (SOD3), in TBI patients. It is a main antioxidant enzyme and proven to protect from brain injury in ischemic stroke.^60^ In rat models, SOD3 overexpression has shown to prevent ischemic–reperfusion injury as well help with regeneration of tissue.^61^

Protease inhibitor, alpha-1-antichymotrypsin (SERPINA3), was upregulated 2–4 times, with the maximum number seen in comparison with the control group. Although serine proteases play a role in normal cellular function, they have been attributed to cell death and apoptosis in the CNS and by inhibiting lead to protective properties.^62,63^ Serine protease and its inhibition have been targeted with virus-derived immune modulators in rats in response to CNS injury, but more research is needed.^64^ Another study administering the SERPINA3 therapeutic agent pointed to interesting effects on inhibiting neuronal death after cerebral ischemia.^65^ Serine protease inhibitors were significantly upregulated in our study.

### Hormone-related proteins and metabolism

Transthyretin (TTR) transport protein is a protein produced in the liver and CSF, carrying retinol and thyroxine as well as causing amyloidosis when misfolding.^66^ TTR was upregulated about 2–2.5 times in the TBI group compared with all other groups. It is a marker of BBB disruption.^67^ This may point to a more extensive damage of the BBB in the TBI patients compared with the other disorders.

Chromogranin A (CHGA) is distributed in secretory vesicles in neurons and neuroendocrine cells. In the presence of microglia shown to induce an inflammatory and neurodegenerative environment.^68^ It also plays a role in reducing BBB damage in septic brain injury.^69^ CHGA was downregulated in TBI patients in comparison with all other groups, why it might be of more importance as a neuroendocrine-immune system component in more infectious-related inflammations in the CNS.^70,71^

Apolipoprotein, APOE, is postulated to play a crucial role in lipid transport within the cerebral environment, contributing to the preservation of microtubular structural integrity within neurons.^72^ In addition, it is involved in neural transmission.^73^ Recent findings from TBI models involving transgenic subjects further substantiate the significance of APOE in modulating both the inflammatory response and neuronal repair mechanisms subsequent to TBI.^72^ APOE protein was significantly downregulated about fivefold in all comparisons, suggesting a dramatic decrease in especially traumatic inflammation disorders. In the cerebrum, astrocyte-sourced APOE assumes a critical function in governing cerebral cholesterol metabolism and facilitating the clearance of β-amyloid.^74^ Noteworthy is that research has revealed an inverse correlation, where reduced concentrations of plasma APOE are concomitant with an escalated susceptibility to dementia.^75,76^ Studies have shown a link between BBB integrity and APOE-protein e4 allele,^77^ but it is not clear in what way and which role the other different polymorphs of the proteins play. One group of researchers discovered an inverse relationship between soluble APOE and BBB leakage, demonstrating a decrease in BBB leakage as APOE levels increase postinjury.^78^ Their cortical punch analysis in mice showed a drastic decrease in local APOE the first day and that it increased after time due to suspected reasons being return of pericytes, decreased matrix-metallopeptidase-9 (MMP-9) expression, and stabilization of tight junctions.^78^

### Inflammatory response

Reactive astrogliosis in TBI has been shown to have both beneficial and problematic effects on the damage exposure to neural tissue.^79^ Identified in astrocytes localized to the traumatic penumbra post-experimental brain contusion, there is an observed upregulation of the glycoprotein, chitinase-3-like protein 1 (CHI3L1). Furthermore, CHI3L1 knockout (KO) mice present with severe astrocytosis and increased microglial/macrophage activity following TBI.^80^ We found 10 times upregulated levels of CHI3L1 in TBI patients compared with the other groups. In recent years, CHI3L1 has garnered attention as an increasingly proposed biomarker with sensitivity and significance, playing a crucial role in the astrocytic response that modulates neuroinflammation.^81^

C3 complement protein showed marked increase in the TBI group compared with the noninfectious inflammatory group (3.5 times), but even higher compared with the control group.^5,6^ C3 was not among the significantly regulated proteins in comparison with the infectious inflammatory group, indicating its central role in different types of CNS injuries. The complement system constitutes a component of the innate immune system, serving as a pivotal player in upholding tissue equilibrium and contributing to the host’s protection against pathogens.^82^ Notably, CSF analyses from TBI patients, spanning various degrees of severity and parenchymal damage, have consistently revealed heightened levels of C3. This elevation exhibits substantial variability both temporally and among patients. Furthermore, a subset of patients displayed intrathecal synthesis of C3.^83^ In contrast, elevated levels of complement factor B (CFB) were observed in the TBI group in comparison with both the noninfectious inflammatory group and the control group. CFB is part of the alternative complement pathway and the amplification loop.^84^ *Postmortem* studies on human TBI cases have identified heightened expression of C3 and CFB in both brain tissue and CSF.^83^ CFB knockout mice demonstrated a decrease in cell death following TBI. This was accompanied by an elevation in antiapoptotic markers and a decrease in proapoptotic markers.^85^ Complement inhibition thus might be a tentative treatment target across various inflammatory disorders. Interestingly, a randomized controlled trial aimed at complement component C1q inhibition following TBI is currently recruiting.^86^

Mucin-like protein IgGFc-binding protein’s (FCGBP) molecular role is still unclear, various studies have revealed that it may be connected to the body’s innate immunity.^87^ In ulcerative colitis, its levels have shown to be downregulated prompting a thinking that it keeps the inflammatory response under control.^88^ On the contrary, a study on gliomas showed high levels of FCGBP to be a poor prognostic factor, and further research is ongoing.^87^ It has been shown that when the FCGBP gene is downregulated, cancer risk is heightened, and FCGBP being a tumor suppressor gene has been suggested.^89^ Of particular significance, elevated concentrations of FCGBP have been identified as contributory factors in the pathogenesis of several neurological disorders characterized by the interplay between inflammation and intestinal dysfunction. Specifically, FCGBP has been associated with amyotrophic lateral sclerosis, where its involvement extends to the facilitation of autoimmune and neuroinflammatory responses.^90^ Furthermore, heightened levels of FCGBP have been observed in Parkinson’s disease, also emphasizing its role as a pertinent neuroinflammatory marker.^91^ In this study, we detected upregulated levels in the TBI group in all three comparisons (2.1, 6.0, and 4.3 times, respectively). To our knowledge, this protein has not before been described in a TBI context.

### Limitations

For all protein quantifications, we utilized mass spectrometry, a method acclaimed for its comprehensive detection of nearly all proteins within samples.^92^ However, a potential limitation lies in the method’s capacity to overshadow less prevalent proteins in normal states, particularly when more abundant counterparts dominate.^93^ Moreover, the number of included patients was modest across all groups, in themselves marked by substantial heterogeneity. In addition, the TBI patients had their CSF drawn from an EVD, while it was obtained by lumbar puncture in controls. It is known that the concentration of some biomarkers differs between the compartments (e.g., S100B, T-tau, and P-tau).^94^ Although this may introduce a potential bias, the procedure of obtaining CSF by lumbar puncture in individuals with an EVD is usually contraindicated. Furthermore, the time point at which the CSF was collected in relation to timing of the trauma was not the same across the groups, although in most cases the time window was restricted to 3 to 7 days. For the groups with inflammatory diseases, drugs given may influence the biomarker concentration. In the Methods section, it is noted if patients received steroids or other immunomodulators.

## Conclusion

In conclusion, contrasting the CSF proteomic profile of victims of TBI recovered in the NICU with HC and individuals suffering from infectious or autoimmune neuroinflammation, we identified unique protein profiles being present in TBI in relation to all comparator groups. The nature of these protein profiles may help identify and classify relevant cellular processes triggered by neurotrauma, in turn the relevance for identifying potentially relevant future therapeutic targets.

## Abbreviations Used

ADEM: acute disseminated encephalomyelitis
BBB: blood–brain barrier
CNS: central nervous system
CSF: cerebrospinal fluid
CXCL: C-X-C motif chemokine ligand
EC: endothelial cells
EVD: external ventricular drain
GCS: Glasgow Coma Scale
GOS: Glasgow outcome score
HC: healthy control
HSE: herpes simplex encephalitis
ICP: intracranial pressure
IFN-: interferon
IL-: interleukin
LFQ: label free quantitation
MS: multiple sclerosis
NICU: neurointensive care unit
NMDAE: anti-*N*-methyl-d-aspartate-receptor encephalitis
RRMS: relapsing–remitting multiple sclerosis
TBI: traumatic brain injury
TNF: tumor necrosis factor
